# Identification and functional analysis of a novel *CSNK2A1* frameshift variant in stillbirth

**DOI:** 10.3389/fgene.2025.1692704

**Published:** 2025-10-27

**Authors:** Nannan Zhang, Miao Han, Tong Zhao, Xinxin Tang, Zhiwei Wang, Yunqiu Du, Leilei Wang

**Affiliations:** ^1^ Department of Prenatal Diagnosis, Lianyungang Maternal and Child Health Hospital of Yangzhou University, Lianyungang, China; ^2^ Laboratory Department, Huaian Blood Center, Huaian, China

**Keywords:** WGS, whole-genome sequencing, CSNK2A1 gene, Stillbirh, intracranial abnormality, gene variant

## Abstract

**Background:**

Casein Kinase II Subunit Alpha (CK2α), the catalytic subunit of protein kinase CK2, is encoded by *CSNK2A1*. This kinase catalyzes substrate phosphorylation and regulates diverse cellular processes including cell cycle progression, apoptosis, and transcription. *CSNK2A1* is associated with Okur-Chung Neurodevelopmental Syndrome (OCNS, OMIM: 617062). Although *CSNK2A1* functional deficiency is implicated in impaired embryonic development, prenatal case reports remain scarce.

**Methods:**

Clinical data and fetal umbilical cord blood samples were collected. Whole-genome sequence (WGS) was used for potential pathogenic variants identification, followed by Sanger sequencing to validate the variant. Bioinformatic tools were employed to predict the 3D structure of the variant. Wild-type and mutant *CSNK2A1* overexpression plasmids were constructed to investigate the functional consequences of the variant.

**Result:**

A 33-year-old pregnant woman without adverse obstetric history. At 34^+4^ weeks, ultrasound showed an intracranial abnormal echoes, multiple cardiovascular anomalies, and stillbirth had occurred at 35 weeks. WGS identified a novel frameshift mutation c.1020_1021delAG (p.Gly342Glnfs*57) in the *CSNK2A1* gene. Bioinformatics analysis indicated structural modification in mutant proteins. *In vitro* kinase assays showed that the variant did not impair kinase activity. Quantitative analysis demonstrated significantly elevated mutant mRNA levels but reduced protein expression compared to wild-type. Elevated ubiquitination in mutants potentially explains diminished CSNK2A1 protein abundance.

**Conclusion:**

We report a novel *CSNK2A1* frameshift mutation that significantly reduces protein expression and impairs gene function. These findings expand our understanding of *CSNK2A1*’s genetic diversity and underscore the importance of comprehensive functional analyses to achieve accurate diagnosis. This study facilitates prenatal diagnosis of *CSNK2A1*-related disorders and informs clinical decision-making for carriers.

## 1 Introduction

Stillbirth is one of the most emotionally devastating pregnancy complications. it is estimated that 13.9 per 1,000 total births or 2.0 million stillbirths at 28 weeks’ gestation or more occur worldwide annually (Hug et al., 2021). Major etiological classifications include obstetric complications, infections, placental insufficiency, intrauterine growth restriction, and congenital abnormalities—with or without identified genetic causes (Lawn et al., 2016). Currently, even with dedicated contemporaneous evaluations, 20%–30% of stillbirth remained without a discernible primary etiology (Page et al., 2017). Genetic factors constitute significant contributors to unexplained stillbirth (Dolanc Merc et al., 2023). Technologies such as whole exome/genome sequencing (WES/WGS), “omics” and functional studies provide avenues to increase our understanding of stillbirth.

Protein kinase CK2 is a ubiquitous serine/threonine kinase with a heterotetrameric configuration (α_2_β_2_, αα′β_2_, or α′_2_β_2_) formed by two catalytic (α/α′) and two regulatory (β) subunits (Wirkner et al., 1998). In addition to their roles in the holoenzyme, all subunits have been proposed to have independent roles in specific tissues. CK2 phosphorylates hundreds of substrates at serine/threonine residues within acidic motifs of enzymes, receptors, transcription factors, and cytoskeletal proteins (Salvi et al., 2009). CK2 is upregulated in cancer cell, and with CK2 inhibitors demonstrating antitumor efficacy (Ruzzene and Pinna, 2010; D’Amore et al., 2020). The catalytic subunit CK2α is encoded by *CSNK2A1* (MIM 115440) on chromosome 20p13. It contains a conserved catalytic domain featuring defined structural motifs: ATP-binding loop, catalytic loop, activation segment, and basic cluster (Unni et al., 2022; Ballardin et al., 2022). *CSNK2A1* is highly expression in the brain, and its knockout induces severe developmental abnormalities in the brain and heart, leading to mid-gestation embryonic lethality (Lou et al., 2008). Studies have shown that CK2α is a modulator of receptor endocytosis and neurotransmitter signaling of Gαs-coupled receptors such as D1, A2a and 5-HT4 receptors (Rebholz et al., 2009; Castello et al., 2017). Drd1a-Cre; CK2α knockout mice display behavioral aberrations: hyperactivity, stereotypy, hyperexploration, impaired motor learning, abnormal nesting, and disrupted circadian rhythms (Rebholz et al., 2013).

In 2016, identified by WES, mutations in *CSNK2A1* were confirmed to be associated with the Okur-Chung neurodevelopmental syndrome (Okur et al., 2016). Typical features of OCNDS observed in children include developmental delays, intellectual impairment, severe brain developmental abnormalities, speech delay, behavioral issues, seizures, and short stature (Jafari Khamirani et al., 2022). Variants can be found along the whole kinase domain of CSNK2A1, and the majority of them are missense mutations. Most mutations affecting the activation loop or ATP-binding domain typically impair kinase activity (Rebholz et al., 2013). In our research, a novel mutation c.1020_1021delAG (p.Gly342Glnfs*57) in the locus on exon 13 of the *CSNK2A1* gene was identified by WGS in a stillbirth case. The C-terminus of CSNK2A1 is an important site of posttranslational modifications and mediates the interaction with peptidyl-prolyl isomerase Pin1 (Messenger et al., 2002). The GST-tagged variants of CK2α Pro231Arg and CK2α Arg312Gln lost 90% of their catalytic activity compared to wild-type (Dominguez et al., 2021). Moreover, mutations near critical residues may disrupt functional motifs; for instance, p.Pro363His could impair CDK1/MAPK1-mediated phosphorylation at Thr360/Ser362 (Ji et al., 2009). The p.Gly342Glnfs*57 mutation in the C-terminal identified in this study has not been reported, and its pathogenicity was still unclear.

To elucidate this mutation’s functional impact and associated fetal developmental pathology, this study presented a bioinformatic analysis and initial functional investigations of the mutation site, providing a pathogenicity assessment through correlation with clinical phenotypic profiles. Our findings aim to expand the genotypic and phenotypic spectrum of *CSNK2A1* and provide new insight its pathogenic mechanism of frameshift variant.

## 2 Materials and methods

### 2.1 Ethical compliance

This study was approved by the Ethics Committee of Lianyungang Maternal and Child Health Hospital (Number: 2024-XM-030). Written informed consent was obtained from the patient for the release of any potentially identifiable image or data contained in this paper.

### 2.2 Whole genome sequencing

Genomic DNA was isolated from fetal umbilical cord blood using nucleic acid extraction kit (MGI Tech, Shenzhen, China) according to the kit instructions. 200–300 ng DNA was used for library construction with the VAHTS Universal DNA Library Prep Kit (Vazyme Biotech Co. Ltd., Nanjing, China). The library construction underwent the following process: end repair, A-tailing, adapter ligation, and PCR amplification. The libraries were sequenced in 150-bp paired-end mode on a DNBSEQ-T7 platform (MGI Tech, Shenzhen, China), with an average coverage depth of >40×. Finally, the original image data were processed through base calling to generate raw sequencing data in FASTQ format. The raw WGS sequencing data had been uploaded to the CNGB Sequence Archive (CNSA) with accession number CNP0008062. Quality-filtered reads were aligned to GRCh37/hg19 using BWA-MEM (v0.7.17) with default parameters. The obtained BAM file was subsequently subjected to variation detection and analysis using GATK (v4.0.11 version). Structural variants were identified using LUMPY (https://github.com/arq5x/lumpy-sv), SNVs and indels were annotated by dbscSNV (http://www.liulab.science/dbscsnv.html), ANNOVAR (https://annovar.openbioinformatics.org/en/latest/). In silico tools, including REVEL, SIFT, Polyphen2, Variant Taster, CADD, and PROVEAN, were employed to predict the pathogenicity of the remaining variants. Disease and phenotype databases, such as OMIM (http://www.omim.org), DECIPHER (https://www.deciphergenomics.org), HGMD (http://www.hgmd.org) and ClinVar (http://www.ncbi.nlm.nih.gov/clinvar) were utilized for further assessment. The candidate variants were classified as pathogenic(P), likely pathogenic (LP), variant of uncertain significance (VUS), benign(B) and likely benign (LB) according to the American College of Medical Genetics and Genomics (ACMG) guidelines (Richards et al., 2015). Sanger sequencing was used for sequence verification.

### 2.3 Structure analysis

The *CSNK2A1* transcript (RefSeq: NM_177,559.2) was obtain from NCBI database (https://www.ncbi.nlm.nih.gov/). Multiple sequence alignment of wild-type and mutant proteins was performed using DNASTAR software (Version: 7.1.0). Protein structures were predicted using AlphaFold2 *via* ColabFold online prediction tool (Version: 1.3) (https://colab.research.google.com/github/sokrypton/ColabFold/blob/main/AlphaFold2.ipynb). Structural alignments were visualized using RCSB protein Data Bank (https://www.rcsb.org/) (Bittrich et al., 2024).

### 2.4 Plasmid construction, cell culture and transfection

Both the wild-type and the mutant *CSNK2A1* gene sequences were custom chemically synthesized, and then cloned into the pCDNA3.1-n3xMyc vector respectively. The recombinant plasmids were subsequently checked by sequencing. The detail information of primers used for plasmid construction and sequence verification were shown in Supplementary Table S1. Positive clones were expanded in *E. coli* DH5α, followed by plasmid extraction. The recombinant plasmids were store at −20 °C for further transfection.

The human neuroblastoma SH-SY5Y cell line was selected for this study because it is a well-established, reproducible, and tractable model system for studying neuronal protein function and regulation. CSNK2A1 is highly expressed in the brain and implicated in neurodevelopment, SH-SY5Y cells provide a relevant cellular context to investigate protein function. SH-SY5Y cells were cultured in 125 μL of DMEM/F12K medium (without antibiotics or serum) at 37 °C/5% CO_2_ and saturated humidity. Cells were seeded in 12-well plates 8 hours prior to transfection. For transfection, 4 μg of plasmid DNA was combined with 4 μL of Lipo8000™ (Beyotime, Shanghai, China) in serum-free medium, followed by gentle pipetting. DNA-lipid complexes were added dropwise to each well according to the specified dosage. Transfection analysis for each sample was conducted in triplicate. After 48 h of incubation, cells were lysed and collected for total RNA and protein extraction.

### 2.5 *In vitro* kinase assay

The *in vitro* kinase assay for mutant CSNK2A1 was performed using UA-Glo ADP glow assay (UA BIOSCIENCE, Nanjing, China), following the manual instructions. Biological triplicates were transfected for each condition. The kinase reaction was performed in universal reaction buffer (40 mM Tris-HCl [pH 7.5], 0.1 mg/mL BSA, 20 mM MgCl_2_) containing substrate peptide (RRRADDSDDDDD). Following the kinase reaction, ATP was removed from the reaction mixture using ATP removal reagent, followed by thorough vortex mixing and incubation at ambient temperature (20 °C–25 °C) for 40 min. Subsequently, kinase ADP detection reagent was added, the mixture was vortexed, and incubated in the dark at ambient temperature for 30 min prior for further fluorescence signal detection.

### 2.6 Quantitative real-time PCR (qRT-PCR) and western blotting (WB) analysis

Total RNA from cells was isolated using the RNeasy kit (TransGen, Biotech, Beijing, China) according to the manufacturer’s recommendations. Quality and quantity were evaluated *via* 1.5% agarose gel electrophoresis and spectrophotometry using Nano ND100. Reverse transcription and cDNA synthesis were performed using HiScript III RT SuperMix (Vazyme, Jiangsu, China) in accordance with the manufacturer’s instructions. Primers were designed using the IDT website (https://sg.idtdna.com/). The mRNA expression level of *CSNK2A1* genes was detected by qRT-PCR Bio-Rad CFX Connect™ Real-Time PCR Detection System (Bio-Rad Laboratories, United States) with the following condition: 95 °C for 5 min pre-incubation, 45 cycles of 95 °C for 15 s and 60 °C for 45 s. The relative quantities of the target genes expressed as fold variation over *GAPDH* were calculated using the 2^−ΔΔCt^ comparative Ct method. Primers used in qRT-PCR was described in Supplementary Table S1.

Cells were harvested and added with RIPA cell lysis buffer (Beyotime, Shanghai, China) containing 1× protease inhibitor (CWBIO, Jiangsu, China) for cell lysis, followed by centrifugation to collect the supernatant. Protein concentrations were quantified using a BCA assay. For sample preparation, lysates were mixed with loading buffer at a 4:1 ratio (v/v) and denatured at 95 °C for 10 min. The samples were separated on 4%–15% Bis-Tris gels and electrophoretically transferred to PVDF membranes. Membranes were blocked with 5% non-fat milk for 2 h at room temperature and incubated overnight at 4 °C with primary antibodies against actin (Proteintech, 66009-1-Ig) and Myc -tag (Proteintech, 16286-1-AP). The membranes were washed three times and then incubated with horseradish peroxidase (HRP)-conjugated goat anti-mouse or anti-rabbit IgG secondary antibody at room temperature for 1 h. After being washed the three times, the membrane was detected with Omni-ECL™ Femto Light Chemiluminescence Kit (epizyme, Shanghai, China) according to the manufacturer’s instructions.

### 2.7 Inhibitor treatment and Co-immunoprecipitation (Co-IP) assay

The SH-SY5Y cells were transferred to a six-well plate and cultured for 48 h. Cell culture and recombinant plasmids transfection adopting the same protocol as mentioned in Section 2.4. MG132 (MCE, Shanghai, China), a specific inhibitor of proteasome, was used to inhibit proteasomal degradation, thereby stabilizing proteins and detect the ubiquitination level of mutant protein. Cells were incubated with MG132 (1 μM) in 6-well plates for 24 h and subsequently harvested. After cell lysis, the samples were divided into two parts. For input group, one part of the sample was used for the protein verification by WB. For IP group, the other part of the sample was added with the anti-Myc working solution pre-treated magnetic beads (Proteintech, Wuhan, China), the mixture was gently shaken at 4 °C overnight. The antigen-antibody-magnetic bead complexes were washed and collected for further WB. The detailed Western blotting procedure followed the protocol described in Section 2.6.

## 3 Results

### 3.1 Clinical presentation

The prenatal diagnostic center received a 33-year-old first pregnant woman with unremarkable obstetric history. The non-consanguineous couple had non-contributory family histories. Routine prenatal surveillance was maintained throughout gestation. The pregnancy progressed without events until 17^+3^ weeks of gestation. At 18 weeks, non-invasive prenatal testing (NIPT) indicated high risk for chromosome 18 anomalies, though concurrent ultrasonography showed no abnormalities. Subsequent karyotype analysis and chromosomal microarray analysis (CMA) returned normal results. Ultrasound at 22^+4^ weeks revealed bilateral choroid plexus cysts that resolved on follow-up imaging, with no other anomalies detected. At 34^+4^ weeks, ultrasound showed an anechoic area posterior to the sixth ventricle midline, intracranial abnormal echoes, and multiple cardiovascular abnormalities. MRI confirmed cranial abnormalities, and stillbirth occurred at 35 weeks. Following multidisciplinary genetic counseling, whole-genome sequencing (WGS) was performed with informed consent.

### 3.2 Identification of a novel *CSNK2A1* frameshift variant

**FIGURE 1:**
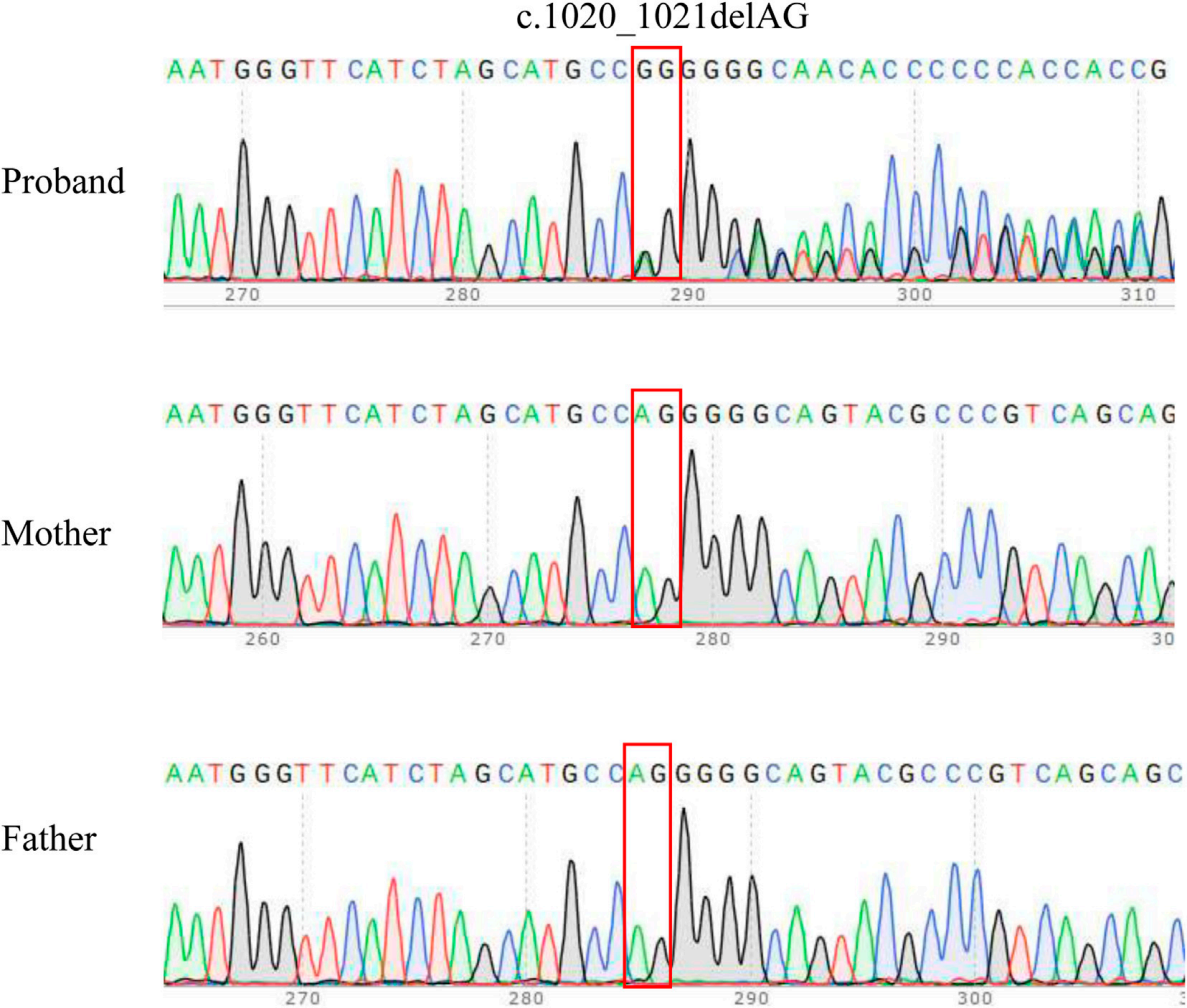
Sanger sequencing results for the proband and the parents. The analysis demonstrated the presence of a frameshift variant in *CSNK2A1* (c.1020_1021delAG) in the proband and the absence of the variant in parents. The red box indicates the variant site.

Whole-genome sequencing (WGS) revealed a novel *CSNK2A1* frameshift variant (NM_177,559.2: c.1020_1021delAG, p.Gly342GlnfsTer57) in the proband. No variants were identified in any of the other established fetal development genes. Thus, variant of CSNK2A1 may be responsible for the phenotype of the proband. Sanger sequencing was used to verify the variant in proband-parent trios (Figure 1). The variant was heterozygous in the proband but absent in both biological parents, confirming *de novo* origin (PS2_Moderate). This variant was absent from population databases (ESP, EXAC and 1000 Genomes Project) and has been undetected in all major control cohorts (PM2_Supporting). The pathogenic mechanism of *CSNK2A1* is loss of function (LOF), while the frameshift mutation did not localize to any major functional domain (PVS1_Moderate). Due to insufficient clinical phenotypic evidence, according to the ACMG variant-interpretation guidelines, this variant was initially classified as VUS (PVS1_M + PS2_M + PM2_P) (Supplementary Table S3). The classification of a VUS indicates that the available evidence is insufficient to determine whether the variant is disease-causing or a harmless polymorphism. As a result, they pose a significant challenge in clinical practice. As evidence accumulates, a proportion of VUS might be reclassified as either (likely) benign or (likely) pathogenic.

### 3.3 Protein sequence and structure analysis

**FIGURE 2:**
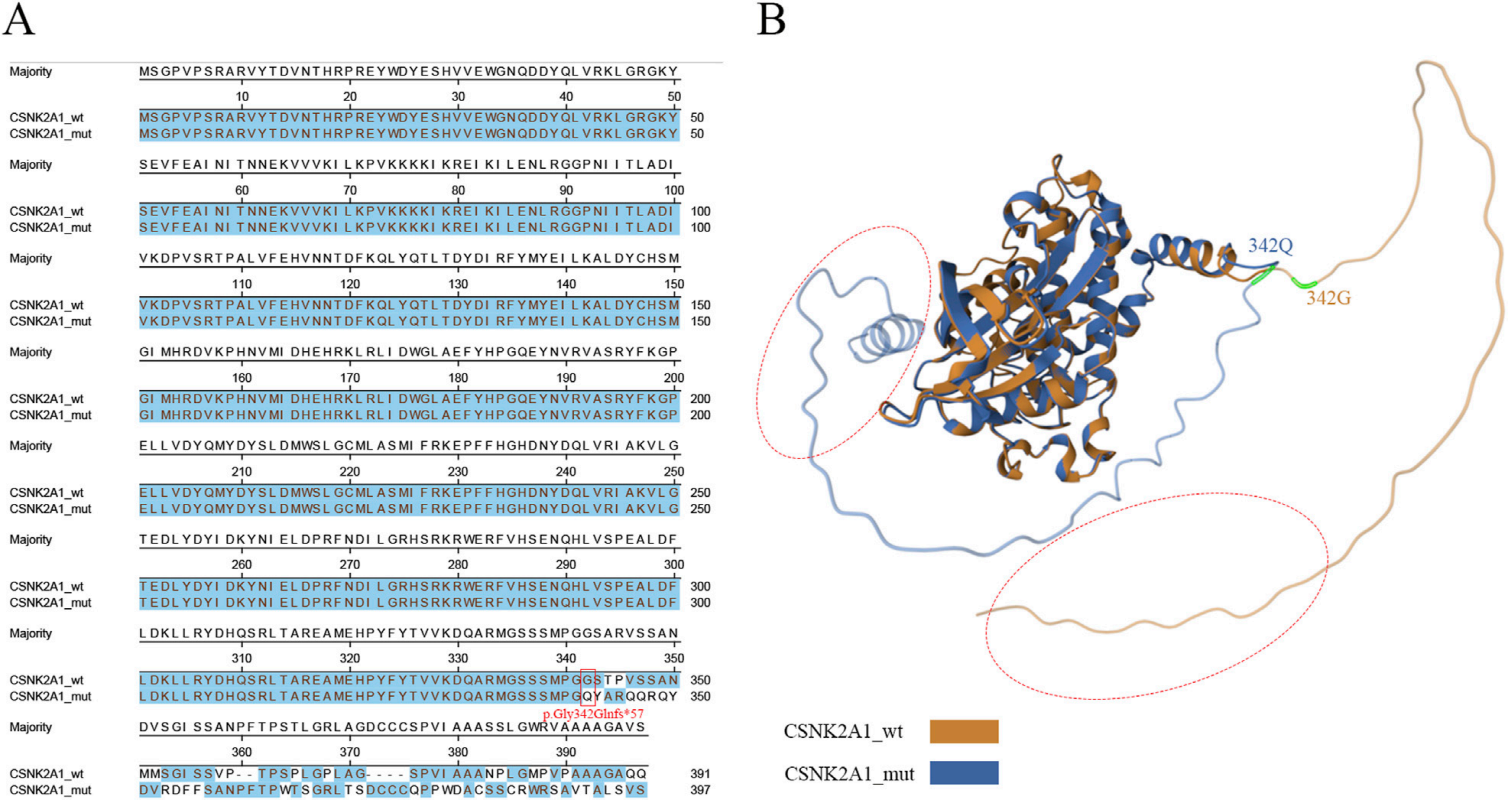
Bioinformatic analysis of wild-type and mutant CSNK2A1. **(A)** The amino acid sequence alignment. The frameshift variant lead to p.Gly342Glnfs*57 in CSNK2A1 is marked in read box, and similar amino acid residues were labeled in blue. **(B)** The comparison between the structure of CSNK2A1 and CSNK2A1’s variants. The wild-type CSNK2A1 is presented in yellow, while the mutant CSNK2A1 is presented in blue color. Red dotted circle: a α-helix is presented in C-terminal of the mutant CSNK2A1, while absent in wild-type.

**FIGURE 3:**
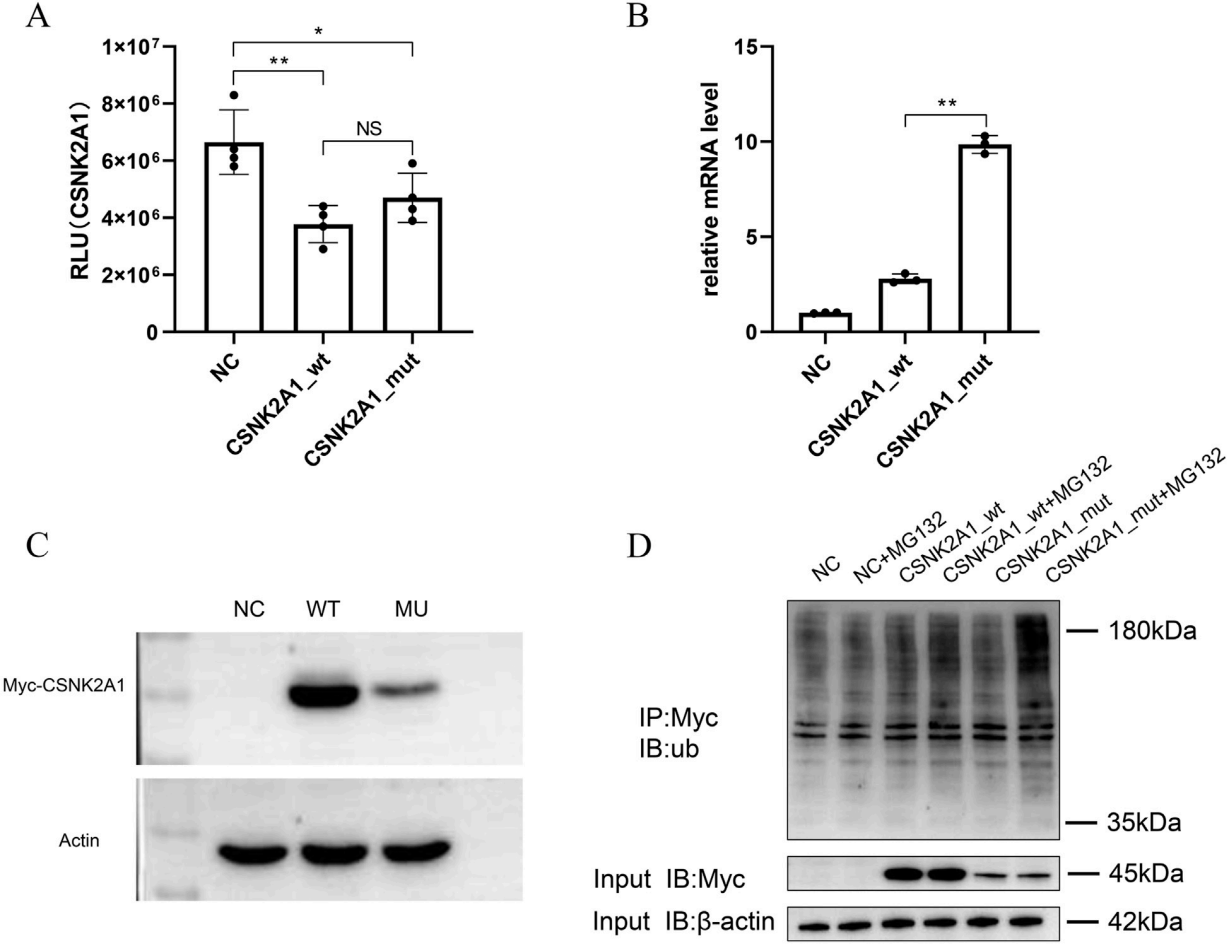
*In vitro* assays of variants in *CSNK2A1* gene. Asterisks ‘**’ and ‘*’indicate statistical significance of *P* < 0.01 and *P* < 0.05 respectively. **(A)** The Kinase activity assays for wild-type and mutant CSNK2A1. No differences were observed between wt and mut group. **(B)** The mRNA expression difference of *CSNK2A1* between wild-type and mutant. The mutant showed a significantly increased mRNA level compared to the wild-type. Asterisks “**” and “*”indicate statistical significance of *P* < 0.01 and *P* < 0.05 respectively. **(C)** The protein expression level Analysis. The CSNK2A1 protein expression level in the mutant was lower than wild-type. **(D)** The ubiquitination levels detection by IP-WB. A significantly higher ubiquitination levels was observed in mutant than in wild-.type.

Compared to the wild-type, the mutant exhibited a frameshift mutation starting at glycine 342 (G342), which replaced the native C-terminal sequence with a novel peptide sequence and caused an extension of six additional residues (Figure 2A). To further investigate potential tertiary structural rearrangements induced by the variant, silico models of wild-type and mutant proteins were generated using AlphaFold2(Version: 1.3) with default parameters. Structural alignment demonstrated a C-terminal α-helical extension in the mutant, while conserved functional domains—including the ATP-binding loop and catalytic loop—retained native conformation (RMSD = 1.21) (Figure 2B). Although not experimentally validated, these predictions provide a mechanistic hypothesis for the observed reduction in mutant protein abundance (Figure 3C), suggesting the C-terminal extension may interfere with protein stability or folding.

### 3.4 Functional characterization of the *CSNK2A1* variant

To assess the functional consequences of *CSNK2A1* (c.1020_1021delAG) variants, the recombined vectors carrying wild-type or mutant *CSNK2A1* genes were constructed and transfected into SH-SY5Y cells. As most pathogenic variants impair catalytic function, *in vitro* kinase activity assays for wild-type and mutant proteins were prioritized. Kinase activity showed no significant difference (*p* > 0.05) between wild-type and p. Gly342Glnfs*57 mutant proteins (Figure 3A), indicating preserved catalytic function and prompting exploration of alternative pathogenic mechanisms. Quantitative analysis revealed divergent mRNA and protein expression: mRNA expression level of mutant *CSNK2A1* was significantly increased (*p* < 0.01) compared to the wild-type (Figure 3B), whereas protein abundance was significantly reduced (*p* < 0.01) (Figure 3C).

The reduced mutant protein abundance suggested enhanced proteasomal degradation. To investigate this, ubiquitination levels were detected by treating with MG132, an inhibitor of proteasome. Result showed that MG132 treatment induced prominent 180 kDa ubiquitin accumulation in mutant-transfected cells compared to wild-type (Figure 3D), demonstrating significantly enhanced ubiquitination. These data indicate that enhanced ubiquitin-mediated degradation likely contributes to the pathogenic mechanism of this *CSNK2A1* variant.

## 4 Discussion

Our study reported a stillbirth case with intracranial anomalies and cardiovascular malformations, where whole-genome sequencing (WGS) identified a novel *CSNK2A1* frameshift variant. Previous studies indicate that even with the presence of congenital anomalies in the fetus, a significant proportion of stillbirth cases have an additional placental finding that could result in fetal death by itself (Page et al., 2017). In this study, placental evaluation of the proband showed no significant abnormalities by morphological examination (Supplementary Figure S1), whereas CNV-seq detected 8%–75% mosaicism across six sites (Supplementary Table S2). Placental mosaicism may disrupt angiogenesis and metabolic homeostasis. Previous studies have demonstrated that placental mosaicism was commonly associated with fetal growth restriction (FGR), with stillbirth occurring in several cases (Taylor et al., 2014; WAPNER and RONALD, 2010). CK2 is essential for placental development, its inhibition impairs trophoblast proliferation, migration, invasion, and syncytialization, compromising feto-maternal circulation (Abi Nahed et al., 2020). We propose that fetal demise resulted from synergistic effects of placental mosaicism and the *CSNK2A1* variant. Due to the intricate pathogenesis of stillbirth, clinical evaluation for unexplained cases should include fetal autopsy, placental pathology examination, and genetic testing.

CSNK2A1 is a catalytic subunit of CK2 holoenzyme, *CSNK2A1* deficiency likely contributes to impaired placental development and adverse fetal outcomes. *De novo* germline variants of the *CSNK2A1* have been reported in individuals with the congenital neuropsychiatric disorder Okur–Chung neurodevelopmental (OCNS) (Chiu et al., 2018; Okur et al., 2016). Most of *CSNK2A1*-related diseases were diagnosed postnatally, while a recent study identified a *CSNK2A1* mutation *via* WES in a prenatal case manifesting fetal brain developmental abnormalities (Kratochwila et al., 2025). In this report, intracranial anomalies and multiple cardiovascular abnormalities were similarly observed. Due to intrauterine fetal demise at 35 weeks, postnatal phenotypic data could not be obtained. Nevertheless, this study expands the documented prenatal phenotypic spectrum associated with *CSNK2A1* mutations, contributing to provide valuable guidance for prenatal diagnostic evaluation.

In this study, a novel frameshift mutation c.1020_1021delAG (p.Gly342Glnfs*57) in *CSNK2A1* gene was identified. The frameshift mutation replaced the native C-terminal sequence with a novel peptide sequence and caused an extension of six additional residues. This variant lack premature termination and localizes outside functional domains; *in vitro* kinase assays confirmed unaffected activity. Previous studies have shown that C-terminal residues of CK2 played a critical role in protein stabilization (Niefind et al., 2001). Our results demonstrated significantly reduced protein levels of the *CSNK2A1* mutant compared to wild-type *in vitro*, suggesting potential involvement of protein degradation pathways. Intracellular proteolysis and protein quality control (PQC) mechanisms are essential prerequisites for maintaining proteome functionality in eukaryotic cells. The protein termini have been recognized as critical indicators for of protein integrity. The unusual amino acid combinations, which indicate damaged or defective proteins was monitored and recognized by PQC (Timms and Koren, 2020; Varshavsky, 2011; Varshavsky, 2019). Recently work indicates that a proteome protection mechanism targets protein with unnatural C terminal sequence by recognizing a surprisingly large number of C-terminal sequence variants (Hasenjäger et al., 2023). Regulated proteolysis of supernumerous or damaged proteins as well as protein aggregates is mainly carried out by the ubiquitin–proteasome system (UPS) and autophagy–lysosome pathways (Finley et al., 2012; Grumati and Dikic, 2018; Ji and Kwon, 2017). Comparative analysis revealed significantly elevated ubiquitination levels in the *CSNK2A1* mutant in this study. We propose that the altered C-terminus compromises stability, inducing ubiquitin-mediated degradation. Whether autophagy participates in mutant protein clearance requires further experimental investigation. While the SH-SY5Y model provided crucial insights into the molecular consequences of the CSNK2A1 mutation, it is important to note its limitations. As a cancer-derived cell line, it may not fully recapitulate the physiological conditions of developing fetal neurons. Future studies would greatly benefit from employing more physiologically relevant models. For example, Patient-derived induced pluripotent stem cells (iPSCs), differentiated into neurons or cerebral organoids, which would capture the patient-specific genetic background and allow for study in a more developmentally accurate context.

In summary, this study, based on bioinformatic analysis and *in vitro* experiments, we confirmed that the mutation c.1020_1021delAG (p.Gly342Glnfs*57) in the locus on exon 13of the *CSNK2A1* gene. The frameshift mutation replaced the native C-terminal sequence with a novel peptide sequence. Although the variant did not impair kinase activity, the modified sequence triggered ubiquitin-mediated degradation. Consequently, reduced protein abundance likely contributes to fetal demise. Our results expanded the spectrum of pathogenic mutations in the *CSNK2A1* gene, and offers theoretical support and research ideas for future studies on related genetic diseases.

## Data Availability

The original contributions presented in the study are publicly available. This data can be found here: https://db.cngb.org/cnsa/ with accession number CNP0008062, and at https://pan.quark.cn/s/16b6de175973.
