# Supplementary material for: Identification and functional analysis of a novel CSNK2A1 frameshift variant in stillbirth
Source: Front Genet. 2025 Oct 27;16:1692704. doi: 10.3389/fgene.2025.1692704 (PMC12597092; doi:10.3389/fgene.2025.1692704)
Supplement: Supplementary file 1 [file DataSheet1.docx]

Table. S1 Primers used in this study

| Primer name | Primer sequence (5’-3’) | usage |
| --- | --- | --- |
| *CSNK2A1*-FW | GGATCCATGTCGGGACCCGTGC | Sequence verification |
| *CSNK2A1*-RV | TCTAGATTACTGCTGAGCGCCA |  |
| 3MYC-*CSNK2A1*-FW | TAATTTCCGAGGAGGACTTAGGATCCATGTCGGGACCCGTGCCAAG | Plasmid construction |
| 3MYC-*CSNK2A1*-RV | CAGCGGGTTTAAACGGGCCCTCTAGATTACTGCTGAGCGCCAGCGGCAG |  |
| qPCR-*CSNK2A1*-FW | AGATGTCAAGCCCCATAATGTCA | qPCR |
| qPCR-*CSNK2A1*-RV | GCCAAACCCCAGTCTATTAGTCG |  |
| *GAPDH*-FW | GGAAGCTTGTCATCAATGGAAATC | Internal reference |
| *GAPDH*-RV | TGATGACCCTTTTGGCTCCC |  |


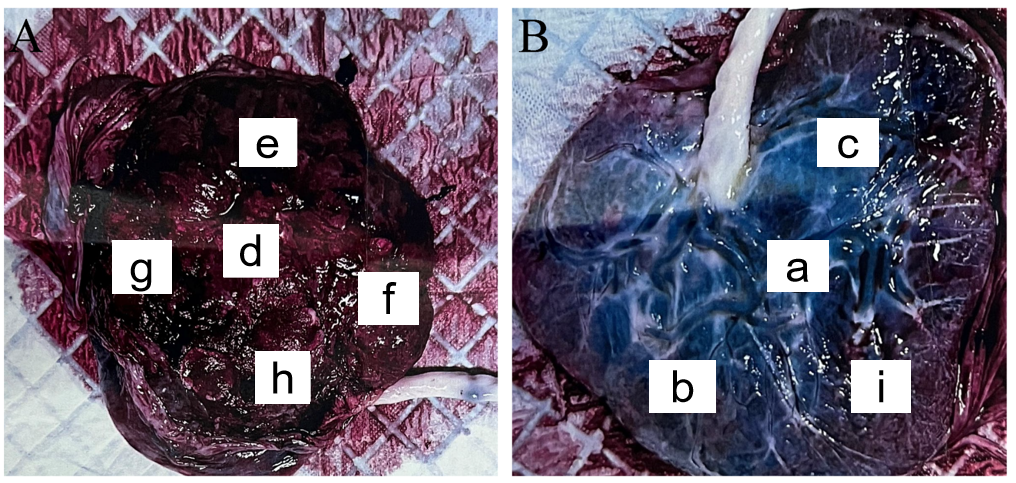


Figure S1: placental morphological examination and samples collection sites for CNV-seq. (A) maternal surface. (B) fetal surface.

**Table S2. CNV-seq results of placental tissue**

| **Sample ID** | **Mosaicism Type** | **Chimeric ratio** |
| --- | --- | --- |
| a | T8 | 8% |
|  | T18 | 60% |
|  | XO | 59% |
| b | T18 | 69% |
|  | XO | 75% |
| c | T18 | / |
|  | T8 | 64% |
|  | XO | 24% |
| d | T18 | 65% |
|  | XO | 69% |
| e | T8 | 43% |
|  | T18 | 60% |
|  | XO | 21% |
| f | T18 | 69% |
|  | XO | 75% |

**Table S3. Summary of ACMG Guidelines Application for the CSNK2A1 variant.**

| ACMG Criterion | Classification Strength | Supporting Evidence from This Study |
| --- | --- | --- |
| PVS1_Moderate | Moderate | Frameshift, Not predicted to undergo NMD, Variant removes < 10% of protein. |
| PS2_ Moderate | Moderate | Denovo origin, No family history, gene-phenotype relationship is not very clear. |
| PM2_Supporting | Supporting | Variant not found in population databases, gene-phenotype relationship is not very clear. |
